# Association between Serum Cystatin C levels and long‐term cardiovascular outcomes and all-cause mortality in older patients with obstructive sleep apnea

**DOI:** 10.3389/fphys.2022.934413

**Published:** 2022-08-31

**Authors:** Jian-Hua Li, Ying-Hui Gao, Xin Xue, Xiao-Feng Su, Huan-Huan Wang, Jun-Ling Lin, Li-Bo Zhao, Xiao Zou, Yan Gao, Jing-Jing Guo, Min Shi, Wei-Hao Xu, Ya-Bin Wang, Xiao-Shun Qian, Kai-Bing Chen, Li Fan, Lin Liu

**Affiliations:** ^1^ Cardiology Department of the Second Medical Center & National Clinical Research Center for Geriatric Diseases, Chinese PLA General Hospital, Beijing, China; ^2^ PKU-UPenn Sleep Center, Peking University International Hospital, Beijing, China; ^3^ Department of Respiratory, Yanan University Affiliated Hospital, Yan’an, China; ^4^ Medical College, Yan’an University, Yan’an, China; ^5^ Department of Respiratory and Critical Care Medicine, Beijing Chaoyang Hospital Affiliated to Capital Medical University, Beijing, China; ^6^ Department of General Practice, 960th Hospital of PLA, Jinan, China; ^7^ Sleep Medicine Center, Department of Respiratory and Critical Care Medicine, Peking University People’s Hospital, Beijing, China; ^8^ Department of Respiratory and Critical Care Medicine of the Second Medical Center & National Clinical Research Center for Geriatric Diseases, Chinese PLA General Hospital, Beijing, China; ^9^ Sleep Center, Affiliated Hospital of Gansu University of Chinese Medicine, Lanzhou, China

**Keywords:** obstructive sleep apnea, cystatin C, cardiovascular outcomes, mortality, older

## Abstract

**Background and Aims:** To investigate the association between obstructive sleep apnea (OSA) severity and baseline serum cystatin C (Cys-C) concentration and to explore the association between baseline serum Cys-C and long-term cardiovascular outcomes and mortality in older patients with OSA.

**Methods:** Between January 2015 and October 2017, a total of 1107 consecutive eligible older patients (≥60 years) with OSA were included in this multicenter, prospective cohort study, and baseline demographics, clinical characteristics, sleep parameters, and follow-up outcomes were collected. Participants were divided into different groups based on baseline serum Cys-C levels. The primary end point was major adverse cardiovascular events (MACE) and the secondary end point was all-cause mortality. The correlation between OSA severity and baseline serum Cys-C was evaluated by Spearman correlation analysis. Multivariate Cox regression was used to analyze the association between Cys-C and the incidence of MACE and mortality.

**Results:** Participants included 672 men and 435 women, with a median age of 66 (range, 60–96) years. At baseline, apnea–hypopnea index (AHI) (*r* = 0.128, *p* < 0.05), oxygen desaturation index (ODI) (*r* = 0.116, *p* < 0.05), and the lowest pulse oxygen saturation (LSpO_2_) (*r* = −0.097, *p* < 0.05) were correlated with serum Cys-C concentration. During the median follow-up period of 42 months, 97 patients (8.8%) experienced MACE and 40 patients (3.6%) experienced death. The association between serum Cys-C levels and the risk of MACE and all-cause mortality was slow rising shaped. The multivariable Cox regression analysis showed patients with a serum Cys-C concentration of ≥1.14 mg/L had higher risks of MACE (HR = 5.30, 95% CI: 2.28–12.30, *p* < 0.05) and all-cause mortality (HR = 9.66, 95% CI: 2.09–44.72, *p* < 0.05) compared with patients with serum Cys-C of ≤0.81 mg/L in older patients with OSA. The receiver-operating characteristic curve showed baseline serum Cys-C levels exhibited moderately capable of identifying patients with a long-term risk of clinical adverse events (MACE and mortality).

**Conclusion:** OSA severity was positively correlated with serum Cys-C concentration. High levels of Cys-C were independently associated with increased risks of MACE and all-cause mortality in older patients with OSA, suggesting that lowering Cys-C levels should be considered as a therapeutic target, and monitoring serum Cys-C may be beneficial to the favorable prognosis of older patients with OSA.

## Introduction

Cystatin C (Cys-C), a member of the superfamily of cysteine protease inhibitors, is expressed in nuclear cells, secreted in body fluids, and involved in the catabolism of intracellular proteins, antigen presentation, apoptosis, etc. (Jiang et al., 2020). It is an effective marker for evaluating kidney function, which is almost completely filtered by glomeruli (Pasala and Carmody, 2017; Raman et al., 2017). Compared with serum creatinine, Cys-C is less affected by age, gender, and weight (Zha et al., 2015). Recently, numerous studies have clarified the relationship between Cys-C and cardiovascular disease risk factors and cardiovascular events in different populations (Angelidis et al., 2013; Ermetici et al., 2015; Chen et al., 2019). A Framingham Offspring Study showed that high Cys-C was independently associated with CVD risk factors even in the absence of chronic kidney disease (CKD) (Parikh et al., 2008). A meta-analysis, mostly derived from high cardiovascular populations, found that Cys-C is strongly and independently associated with subsequent CVD risk. The highest Cys-C category versus the lowest was associated with a subsequent greater risk of CVD, coronary heart disease, and stroke (Lee et al., 2010). The imbalanced expression between cysteine protease and Cys-C is currently thought to be one of the reasons for the progression of cardiovascular diseases such as atherosclerosis and aneurysms (Sukhova et al., 2005; Bengtsson et al., 2008). However, other studies did not support the causal role of Cys-C in the etiology of CVD (van der Laan et al., 2016).

Obstructive sleep apnea (OSA), a common sleep disorder, is characterized by recurrent episodes of upper airway collapse during sleep, leading to decreased oxygen saturation and sleep fragmentation (Zhang and Veasey, 2012). Studies have shown that OSA is one of the independent risk factors for a variety of chronic systemic diseases, such as hypertension, coronary heart disease, stroke, etc. (Bouzerda, 2018; Gabryelska et al., 2018). OSA has been associated with the pathophysiology of cardiovascular disease via multiple pathological mechanisms, such as intermittent hypoxia, vascular endothelial dysfunction, various autonomic changes, activation of the renin–angiotensin system, oxidative stress, inflammatory reactions, and promoting atherosclerosis (Salman et al., 2020).

A previous cross-sectional study indicated that severe OSA independently increased serum Cys-C levels in patients without CKD (Kato et al., 2011). In younger men, Cys-C was correlated with apnea–hypopnea index (AHI), oxygen desaturation index (ODI), hs-CRP, creatinine, and estimated glomerular filtration rate (Zhang et al., 2013). Due to a greater tolerance to hypoxia in older people, the association between the severity of OSA and serum Cys-C concentration may be different from that in young patients (Lavie and Lavie, 2006). To the best of our knowledge, it is still not clear whether patients with OSA and increased serum Cys-C levels are prone to a long-term risk of cardiovascular events, especially in older patients.

Therefore, we performed a large-scale, multicenter, prospective cohort study and did a survival analysis to delineate the association of OSA severity and baseline serum Cys-C concentration with subsequent cardiovascular outcomes and mortality in older patients with OSA.

## Materials and methods

### Study population

A total of 1290 elderly patients with OSA were recruited from the PLA General Hospital (*n* = 313), Peking University International Hospital (*n* = 238), Peking University People’s Hospital (*n* = 242), Beijing Chaoyang Hospital (*n* = 337), the 960th Hospital of PLA (*n* = 48), and the affiliated Hospital of Gansu University of Chinese Medicine (*n* = 112) from January 2015 to October 2017 as described in our previous study (Su et al., 2021a). Inclusion criteria were as follows: 1) age ≥60 years, 2) compliance with OSA diagnostic criteria, 3) willingness to participate in this study, and signed informed consent. We excluded 166 patients based on the following criteria: 1) previous history of myocardial infarction (MI), hospitalization for unstable angina and heart failure (*n* = 34); 2) receiving any treatment for OSA (*n* = 76); 3) patients with malignant tumors (*n* = 3); 4) patients with mental disorders (*n* = 4); 5) presence of kidney diseases (*n* = 48); and 6) incomplete data of serum Cys-C (*n* = 1). Furthermore, we excluded 17 patients who had lost their visit during the follow-up. Ultimately, 1107 patients with OSA were included in this data analysis. For the purpose of our study, patients were divided into different groups based on baseline serum Cys-C levels among elderly OSA. The Ethics Committee of PLA General Hospital approved the study (S2019-352-01).

### PSG examination

Sleep parameters of all patients were recorded using a laboratory‐based polysomnography (PSG) instrument (Compumedics, Melbourne, Australia) as described previously (Qian et al., 2012). All participants were continuously monitored for more than 7 h. The PSG consisted of continuous polygraphic recording from surface leads for electroencephalography, electrooculography, electromyography, electrocardiography, thermistors for nasal and oral airflow, thoracic and abdominal impedance belts for respiratory effort, pulse oximetry for oxyhemoglobin concentration, a tracheal microphone for snoring, and a sensor for the position during sleep. PSG records were staged manually according to standard criteria.

### Definitions

Sleep parameters: Apnea was defined as the continuous cessation of airflow for more than 10 s, whereas hypopnea was defined as a 30% or greater drop inflow for 10 s or longer, associated with ≥4% oxygen desaturation (Berry et al., 2012). The AHI) was defined as the number of apnea and hypopnea events per hour during sleep. The ODI was defined as the number of ≥3% arterial oxygen desaturations per hour of sleep. T90 was the percentage of the times for oxygen saturation (SaO_2_) <90% in the total monitoring time during overnight sleep. The lowest pulse oxygen saturation (LSpO_2_), the mean pulse oxygen saturation (MSpO_2_), the mean apnea time (MAT), and the longest apnea time (LAT) were recorded during overnight sleep. OSA severity was defined according to AHI based on the criteria of the American Academy of Sleep Medicine as follows (Berry et al., 2012): mild OSA, 5 ≤ AHI <15; moderate OSA, 15 ≤ AHI <30; and severe OSA, AHI ≥30.

Comorbidities: Hypertension was defined as the mean of at least two consecutive measurements of SBP/DBP was ≥140/90 mmHg or recieving antihypertensive therapy (Ma et al., 2021). Dyslipidemia was defined based on Chinese Guideline for the management of hyperlipidemia in adults (Joint committee issued Chinese guideline for the management of dyslipidemia in adults, 2016). Coronary heart disease (CHD), carotid atherosclerosis, and chronic obstructive pulmonary disease (COPD) were determined by a record of a relevant diagnostic clinical (Read) code indicating the presence of the condition (Charlson et al., 1987). Atrial fibrillation was defined based on the AHA/ACC/HRS 2014 guidelines (January et al., 2014).

### Procedures and management

All patients received standard health care according to their different basic diseases during follow-up. We recommended that patients with moderate to severe OSA go to a sleep center for treatment of OSA.

### Follow-up

The follow-up period started at the time of PSG assessment and ended at the end of the study period (December 2020). The participants’ outcomes were collected by clinic visits or telephone calls by an investigator who was blinded to the patients’ PSG results every 6 months.

The primary end point was major adverse cardiovascular events (MACE), defined as a composite of cardiovascular death, MI, and hospitalization for unstable angina or heart failure. The secondary end point was all-cause mortality.

### Statistical analysis

Statistical analysis was performed using SPSS version 20.0 (SPSS Inc., Chicago, Illinois, United States). Categorical variables are presented as counts (percentages) and compared using Chi-square tests. Normally distributed continuous variables are presented as mean ± standard deviation (SD) and compared using the *t*-test. Skewed variables are presented as medians (interquartile range) and compared using the Kruskal–Wallis Rank Sum-test. The correlation between sleep parameters and baseline serum Cys-C was analyzed using Spearman correlation analysis. The unadjusted Kaplan–Meier curve was used to visualize the association of baseline serum Cys-C and the adverse events. A Cox proportional hazard regression analysis was performed to assess hazard ratios for MACE and all-cause mortality according to different levels of serum Cys-C in older patients with OSA. Model 1 was unadjusted. In model 2, analyses were partially adjusted for age, sex, and BMI. Model 3 takes into account all potential confounders factors, including age, sex, BMI, SBP, creatinine, uric acid, AHI, ODI, LSpO_2_, LAT, CHD, hypertension, hyperlipidemia, diabetes, atrial fibrillation, and carotid atherosclerosis. The associations between levels of serum Cys-C and all endpoints were evaluated on a continuous scale with restricted cubic spline curves based on Cox proportional hazards models. The receiver-operating characteristic curve was plotted to estimate the capability of serum Cys-C to discriminate against patients with all endpoints. The results were reported as hazard ratios (HRs) together with their 95% confidence intervals (CI).

## Results

### Baseline characteristics

**FIGURE 1:**
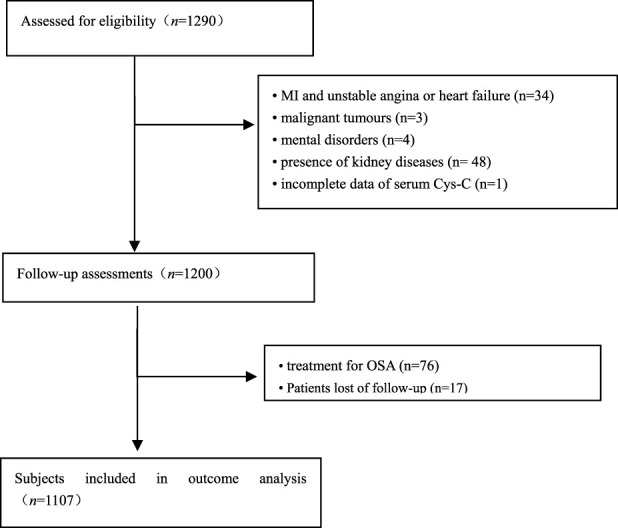
Flowchart of participant selection. OSA: obstructive sleep apnea; MI: myocardial infarction.

In total, 1290 consecutive eligible patients with OSA were prospectively enrolled, all of whom underwent a successful overnight sleep study. After the exclusion of patients according to predefined criteria, 1107 patients were included in the final analysis (Figure 1). Participants included 672 men and 435 women, with a median age of 66 (range, 60–96) years. Table 1 shows the baseline characteristics of enrolled patients according to quartile of serum Cys-C concentration. Age, sex, BMI, SBP, BUN, creatinine, uric acid, HbA1c, HDL-C, AHI, ODI, LSpO_2_, LAT, CHD, hypertension, hyperlipidemia, diabetes, atrial fibrillation, and carotid atherosclerosis were significantly different between these four categories (*p* < 0.05). There was no significant difference in other listed variables between the groups.

**TABLE 1:** Characteristics of elderly OSA participants according to quartiles of serum Cys-C.

	Quartile 1 (*n* = 277)	Quartile 2 (*n* = 281)	Quartile 3 (*n* = 285)	Quartile 4 (*n* = 264)	*p*-Value
Age,y	65 (62, 69)	65 (62, 70)	66 (63, 71)	67 (62, 74)	0.005
Male,n (%)	141 (49.8)	168 (60.4)	173 (61.8)	190 (71.4)	0.000
BMI(kg/m^2^)	26.10 (23.70, 28.28)	25.83 (23.66, 28.07)	26.62 (24.00, 29.07)	26.71 (24.34, 30.00)	0.005
SBP (mmHg)	130 (120, 140)	130 (124, 140)	130 (123, 140)	136 (126, 150)	0.005
DBP (mmHg)	76 (69, 83)	76 (70, 82)	76 (70, 82)	80 (70, 85)	0.221
Smoking,n (%)	44 (15.9)	41 (14.6)	38 (13.3)	37 (14.0)	0.852
Drinking,n (%)	20 (7.2)	17 (6.0)	29 (10.2)	32 (12.1)	0.051
ALT, U/L	16.6 (12.0, 26.4)	16.8 (12.0, 25.1)	17.1 (12.7, 25.8)	17.8 (12.7, 28.3)	0.794
AST, U/L	18.0 (14.0, 23.0)	17.3 (14.5, 22.5)	17.5 (14.6, 23.0)	17.4 (14.1, 23.0)	0.924
BUN, mmol/L	6.1 (5.0, 8.8)	6.2 (5.2, 8.0)	6.1 (4.9, 8.2)	6.7 (5.0, 9.8)	0.032
Creatinine, umol/L	67.7 (57.8, 78.0)	71.0 (61.4, 79.9)	73.3 (61.4, 83.0)	78.4 (68.0, 98.1)	0.000
Uric acid, umol/L	323.0 (260.0, 370.0)	340.0 (306.7, 380.0)	345.0 (312.0, 390.0)	389.0 (332.2, 443.0)	0.000
HbA1c, %	5.52 (5.08, 5.52)	5.52 (5.30, 5.52)	5.52 (5.16, 5.52)	5.41 (4.96, 5.74)	0.036
TG, mmol/L	1.39 (0.99, 1.87)	1.35 (1.01, 1.94)	1.29 (0.99, 1.84)	1.45 (1.02, 1.93)	0.400
TC, mmol/L	4.19 (3.49, 5.06)	4.21 (3.59, 4.99)	4.25 (3.57, 4.94)	4.27 (3.57, 4.92)	0.998
LDL-C, mmol/L	2.52 (1.88, 2.99)	2.47 (1.91, 3.03)	2.41 (1.88, 3.09)	2.36 (1.84, 2.93)	0.572
HDL-C, mmol/L	1.10 (0.88, 1.32)	1.12 (0.92, 1.42)	1.12 (0.91, 1.43)	1.03 (0.87, 1.30)	0.012
AHI, events/h	23.8 (12.6, 37.3)	25.4 (14.3, 44.8)	26.5 (15.1, 45.2)	33.4 (17.9, 49.5)	0.000
ODI, events/h	18.3 (8.7, 34.8)	22.0 (11.1, 39.9)	19.4 (10.2, 39.0)	25.9 (11.5, 46.4)	0.001
MSpO_2_, %	94 (92, 95)	93 (92, 95)	93 (92, 95)	93 (91, 95)	0.252
LSpO_2_, %	82 (75, 86)	81 (73, 85)	80 (73, 85)	79 (69, 85)	0.029
MAT, s	22.6 (19.5, 25.1)	22.9 (20.0, 25.5)	22.4 (18.7,25.7)	22.2 (19.2,25.7)	0.614
LAT, s	57.0 (36.0, 83.2)	67.0 (45.0, 88.0)	61.8 (41.8, 85.0)	65.0 (43.5, 85.2)	0.049
T90, %	2.87 (0.52, 14.03)	3.71 (0.65, 15.25)	3.50 (0.58,16.27)	4.00 (0.62,14.71)	0.839
CHD, n (%)	44 (15.9)	64 (22.8)	61 (21.4)	75 (26.3)	0.005
Hypertension, n (%)	154 (55.6)	182 (64.8)	177 (62.1)	187 (70.8)	0.003
Hyperlipidemia, n (%)	73 (26.4)	69 (24.6)	77 (27.0)	90 (34.1)	0.001
Diabetes, n (%)	51 (18.4)	69 (24.6)	56 (19.6)	85 (32.2)	0.000
AF, n (%)	11 (4.0)	21 (7.5)	20 (7.0)	35 (13.3)	0.000
Paroxysmal AF, n (%)	7 (2.5)	14 (5.0)	14 (4.9)	24 (9.1)	0.000
Persistent AF, n (%)	4 (1.4)	7 (2.5)	6 (2.1)	11 (4.2)	0.000
Carotid atherosclerosis, n (%)	66 (23.8)	55 (19.6)	72 (25.3)	88 (33.3)	0.003
COPD, n (%)	19 (6.9)	12 (4.3)	19 (6.7)	26 (9.8)	0.084

Quartile 1: Cys-C ≤ 0.81 mg/L; Quartile 2: 0.81<Cys-C≤0.96 mg/L; Quartile 3: 0.97≤Cys-C≤1.13 mg/L; Quartile 4: Cys-C≥1.14 mg/L. BMI: body mass index; SBP: systolic blood pressure; DBP: diastolic blood pressure; ALT: alanine aminotransferase; AST: aspartate aminotransferase; BUN: blood urea nitrogen; TG: triglyceride; TC: total cholesterol; LDL-C: low density lipoprotein cholesterol; HDL-C: high density lipoprotein cholesterol; AHI: the apnea-hypopnea index; ODI: the oxygen desaturation index; MSpO2: the mean pulse oxygen saturation; LSpO2: the lowest pulse oxygen saturation; MAT: the mean apnea time; LAT: the longest apnea time; T90: percentage of the times for SaO2<90% in total monitoring time during overnight sleep; OSA: obstructive sleep apnea; CHD: coronary heart disease; AF: atrial fibrillation; COPD: chronic obstructive pulmonary disease.

### Correlations between sleep parameters and serum Cys-C concentration

Compared with the participants in Quartile 1 (Cys-C ≤ 0.81 mg/L), those in Quartile 4 (Cys-C ≥ 1.14 mg/L) tended to have higher AHI, ODI, LAT, and lower LSpO_2_ (*p* < 0.05; Table 1). We further examined the association between sleep parameters and serum Cys-C levels. The Spearman correlation analysis showed that AHI (*r* = 0.128, *p* < 0.05) and ODI (*r* = 0.116, *p* < 0.05) were positively correlated with serum Cys-C concentration (Supplementary Figure S1). In contrast, there was a week negative correlation between LSpO_2_ and serum Cys-C concentration (*r* = −0.097, *p* < 0.05; Table 2, Supplementary Figure S1).

**TABLE 2:** Correlations between sleep parameters and Cys-C levels.

	**Spearman correlation**	
	***r***	***p*-Value**
AHI	0.128	0.000
ODI	0.116	0.000
MSpO_2_	−0.050	0.098
LSpO_2_	−0.097	0.001
MAT	−0.022	0.455
LAT	0.034	0.256
T90	0.030	0.325

AHI: the apnea-hypopnea index; ODI: the oxygen desaturation index; MSpO_2_: the mean pulse oxygen saturation; LSpO_2_: the lowest pulse oxygen saturation; MAT: the mean apnea time; LAT: the longest apnea time; T90: percentage of the times for SaO2<90% in total monitoring time during overnight sleep.

### Association between Cys-C and the risk of clinical outcomes during follow-up primary end point: MACE

Table 3 shows the crude numbers of adverse events. MACE developed during the median follow-up period of 42 months (range: 1–72 months) in 97 patients (8.8%). The cumulative incidence rates of MACE within the follow-up period among elderly patients with OSA in the four quartiles of serum Cys-C (from low to high) were 2.5, 5.0, 11.2, and 16.7%, respectively.

**TABLE 3:** Crude number of clinical outcomes during follow-up.

Follow-up outcomes	Quartile1 (*n* = 277)	Quartile 2 (*n* = 281)	Quartile 3 (*n* = 285)	Quartile 4 (*n* = 264)
MACE,n (%)	7 (2.5)	14 (5.0)	32 (11.2)	44 (16.7)
Cardiovascular death,n (%)	0 (0.0)	1 (0.4)	6 (2.1)	13 (4.9)
MI,n (%)	1 (0.4)	5 (1.8)	6 (2.1)	14 (5.3)
Hospitalization for unstable angina,n (%)	5 (1.8)	10 (3.6)	22 (7.7)	19 (7.2)
Hospitalization for heart failure,n (%)	1 (0.4)	0 (0.0)	3 (1.1)	6 (2.3)
All-cause mortality, n (%)	2 (0.7)	6 (2.1)	13 (4.6)	19 (7.2)

MACE: major adverse cardiovascular events; MI: myocardial infarction.

**FIGURE 2:**
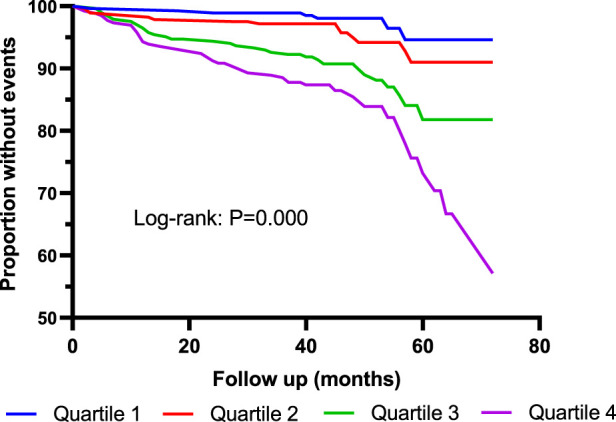
Kaplan–Meier curves for MACE according to quartiles of serum Cys-C. Log-rank test: *p* = 0.000 (Quartile 1 vs. Quartile 4). MACE: major adverse cardiovascular events. Quartile 1: Cys-C ≤ 0.81 mg/L; Quartile 2: 0.81<Cys-C≤0.96 mg/L; Quartile 3: 0.97≤Cys-C≤1.13 mg/L; Quartile 4: Cys-C≥1.14 mg/L.

**FIGURE 3:**
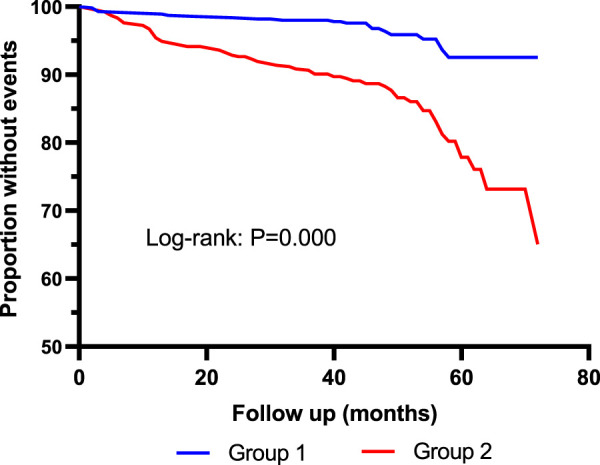
Kaplan–Meier curves for MACE according to median of serum Cys-C. Log-rank test: *p* = 0.000. Group 1: Cys-C≤0.96 mg/L; Group 2: Cys-C≥0.97 mg/L.

Kaplan–Meier curves showed that the incidence of MACE was significantly higher in Quartile 4 (Cys-C ≥ 1.14 mg/L) patients than in Quartile 1 (Cys-C ≤ 0.81 mg/L) (log-rank test: *p* = 0.000, Figure 2). In another view, the incidence of MACE was significantly higher in patients with Cys-C ≥ 0.97 mg/L than patients with Cys-C ≤ 0.96 mg/L (log-rank test: *p* = 0.000, Figure 3). A multivariate Cox regression analysis was performed to better assess the risk of MACE. After adjustment for age, sex, BMI, SBP, creatinine, uric acid, AHI, ODI, LSpO_2_, LAT, CHD, hypertension, hyperlipidemia, diabetes, atrial fibrillation, and carotid atherosclerosis, the fully adjusted Cox analysis showed that patients in Quartile 4 had a 5.30-fold higher risk of developing MACE compared with those in Quartile 1 (HR = 5.30; 95% CI, 2.28, 12.30; *p* = 0.000; Table 4). Similarly, adjusted HRs for MACE in Group 2 (Cys-C ≥ 0.97) were significantly higher than in Group 1 (Cys-C ≤ 0.96 mg/L) (HR = 3.22; 95% CI, 1.93, 5.36; *p* = 0.000; Table 5).

**TABLE 4:** HRs and 95% CIs of clinical outcomes according to quartiles of serum Cys-C.

	Quartile 1		Quartile 2		Quartile 3		Quartile 4	
	HR (95%CI)	*p*-Value	HR (95%CI)	*p*-Value	HR (95%CI)	*p*-Value	HR (95%CI)	*p*-Value
MACE								
Model 1	1.00 (ref.)	—	1.79 (0.72, 4.44)	0.209	4.33 (1.91, 9.80)	0.000	6.90 (3.10, 15.34)	0.000
Model 2	1.00 (ref.)	—	1.68 (0.68, 4.17)	0.263	3.91 (1.73, 8.90)	0.001	5.86 (2.62, 13.13)	0.000
Model 3	1.00 (ref.)	—	1.69 (0.67, 4.27)	0.264	3.84 (1.66, 8.85)	0.002	5.30 (2.28, 12.30)	0.000
All-cause mortality								
Model 1	1.00 (ref.)	—	2.57 (0.52, 12.73)	0.249	5.59 (1.26, 24.78)	0.024	9.63 (2.24, 41.45)	0.002
Model 2	1.00 (ref.)	—	2.46 (0.50, 12.21)	0.272	4.43 (0.99, 19.78)	0.051	7.99 (1.83, 34.76)	0.006
Model 3	1.00 (ref.)	—	2.55 (0.50, 13.02)	0.261	4.86 (1.05, 22.47)	0.043	9.66 (2.09, 44.72)	0.004

Quartile 1: Cys-C ≤ 0.81 mg/L; Quartile 2: 0.81<Cys-C≤0.96 mg/L; Quartile 3: 0.97≤Cys-C≤1.13 mg/L; Quartile 4: Cys-C≥1.14 mg/L.

Model 1: unadjusted for the Cys-C, group.

Model 2: adjusted for the Cys-C, group, age, sex and BMI.

Model 3:adjusted for the Cys-C, group, age, sex, BMI, SBP, creatinine, uric acid, AHI, ODI, LSpO2, LAT, CHD, hypertension, hyperlipidemia, diabetes, atrial fibrillation and carotid atherosclerosis.

**TABLE 5:** HRs and 95% CIs of clinical outcomes according to median of serum Cys-C.

Group	Group 1	*p*-Value	Group 2	*p*-Value
	HR (95%CI)		HR (95%CI)	
MACE				
Model 1	1.00 (ref.)	—	3.88 (2.39, 6.30)	0.000
Model 2	1.00 (ref.)	—	3.50 (2.15, 5.70)	0.000
Model 3	1.00 (ref.)	—	3.22 (1.93, 5.36)	0.000
All-cause mortality				
Model 1	1.00 (ref.)	—	4.02 (1.85, 8.75)	0.000
Model 2	1.00 (ref.)	—	3.32 (1.52, 7.26)	0.003
Model 3	1.00 (ref.)	—	3.57 (1.56, 8.17)	0.003

Group 1: Cys-C≤0.96 mg/L; Group 2: Cys-C≥0.97 mg/L.

Model 1: unadjusted for the Cys-C, group.

Model 2: adjusted for the Cys-C, group, age, sex and BMI.

Model 3:adjusted for the Cys-C, group, age, sex, BMI, SBP, creatinine, uric acid, AHI, ODI, LSpO2, LAT, CHD, hypertension, hyperlipidemia, diabetes, atrial fibrillation and carotid atherosclerosis.

After adjusting for all potential confounders in model 3, restricted cubic splines showed a slow rising curved association between Cys-C concentration and the risk of MACE (Figure 6). According to the receiver-operating characteristic curve, baseline serum Cys-C concentration was moderately capable of identifying patients with a long-term risk of MACE (AUC = 0.701; 95% CI: 0.647–0.755, *p* = 0.000; Figure 7).

### Secondary end point: All-cause mortality

**FIGURE 4:**
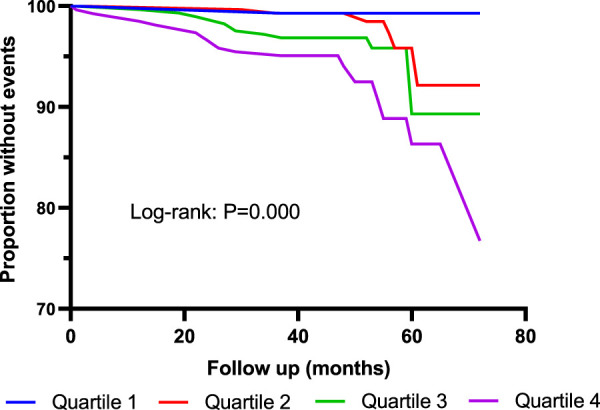
Kaplan–Meier curves for all-cause mortality according to quartiles of serum Cys-C. Log-rank test: *p* = 0.000 (Quartile 1 V S. Quartile 4). Quartile 1: Cys-C ≤ 0.81 mg/L; Quartile 2: 0.81<Cys-C≤0.96 mg/L; Quartile 3: 0.97≤Cys-C≤1.13 mg/L; Quartile 4: Cys-C≥1.14 mg/L.

**FIGURE 5:**
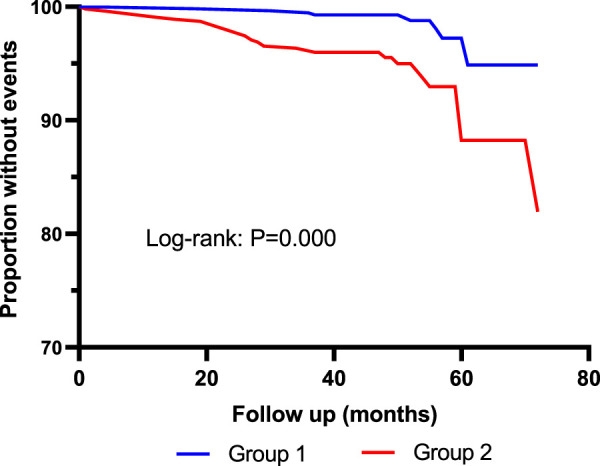
Kaplan–Meier curves for all-cause mortality according to median of serum Cys-C. Log-rank test: *p* = 0.000. Group 1: Cys-C≤0.96 mg/L; Group 2: Cys-C≥0.97 mg/L.

As shown in Table 3, 40 patients died during the follow-up period. Incidence of all-cause mortality was 0.7% in Quartile 1 (Cys-C ≤ 0.81 mg/L) patients, 2.1% in Quartile 2 (0.81< Cys-C ≤ 0.96 mg/L) patients, 4.6% in Quartile 3 (0.97 ≤ Cys-C ≤ 1.13 mg/L) patients, and 7.2% in Quartile 4 (Cys-C ≥ 1.14 mg/L) patients, which showed an upward trend in a dose-dependent manner according to the baseline concentration of serum Cys-C. The fully adjusted Cox analysis showed that Quartile 4 patients had a 9.66-fold higher risk of all-cause mortality compared with those in Quartile 1 (HR = 9.66; 95% CI, 2.09, 44.72; *p* = 0.004; Table 4). Similarly, the risk of all-cause mortality was significantly higher in Group 2 than in Group 1 (HR = 3.57; 95% CI, 1.56, 8.17; *p* = 0.003; Table 5). For different views, Kaplan–Meier curves were used to present the association between baseline serum Cys-C and the risk of all-cause mortality (log-rank test: *p* = 0.000) (Figures 4, 5).

**FIGURE 6:**
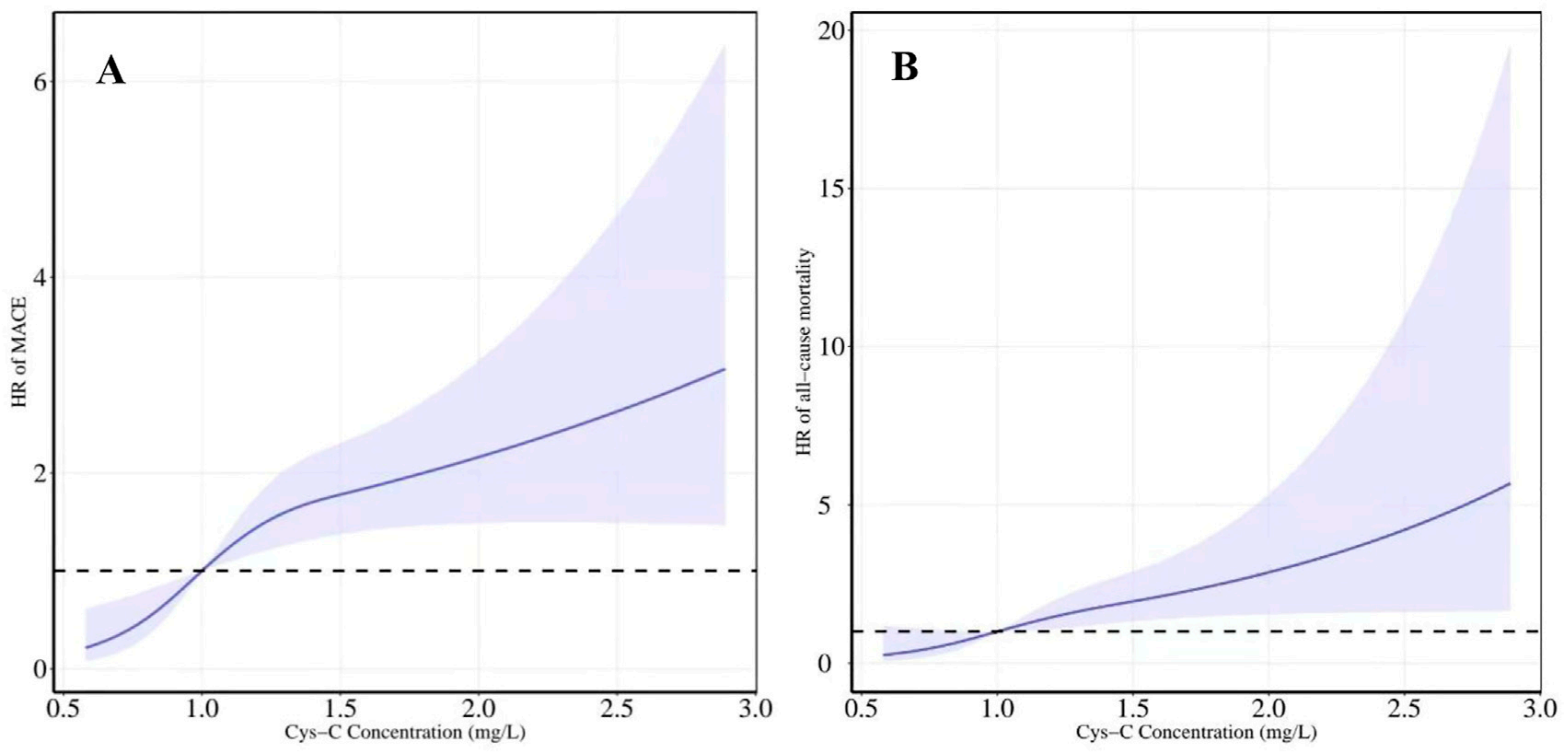
Restricted cubic spline regression model was conducted with three knots at the 10th, 50th, and 90th percentiles of Cys-C. The dotted lines represent the 95% confidence intervals for the spline model (reference is 1.0 mg/L). **(A)** HR for MACE according to levels of Cys-C on a continuous scale **(B)** HR for all-cause mortality according to levels of Cys-C on a continuous scale. Analyses were adjusted for age, sex, BMI, SBP, creatinine, uric acid, AHI, ODI, LSpO2, LAT, CHD, hypertension, hyperlipidemia, diabetes, atrial fibrillation and carotid atherosclerosis. HR indicates hazard ratio.

**FIGURE 7:**
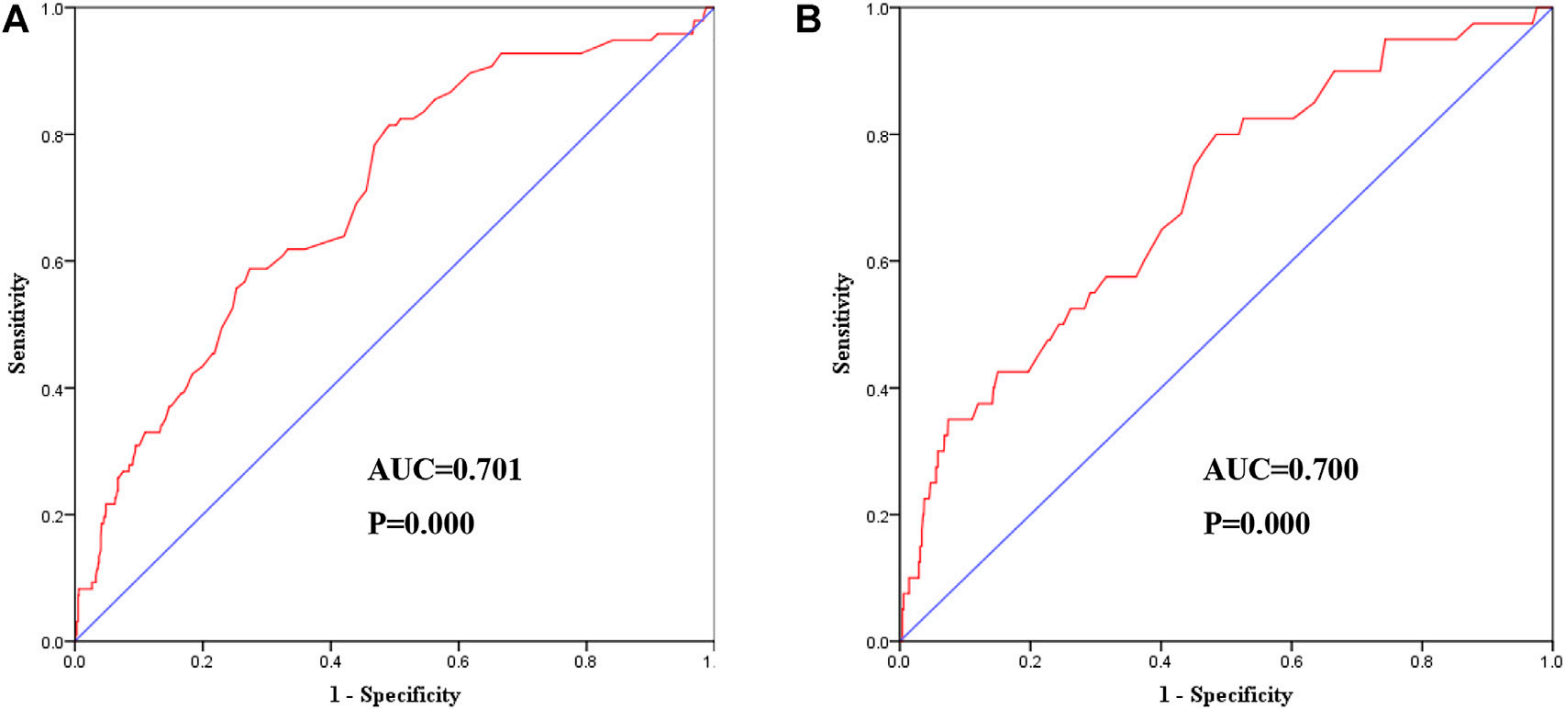
Receiver-operating characteristic curve showed the performance of Cys-C in predicting a high risk of clinical outcomes. **(A)** The AUC was 0.701 (95% CI:0.647–0.755, *p* = 0.000) in predicting a high risk of MACE. **(B)** The AUC was 0.700 (95% CI:0.617–0.784, *p* = 0.000) in predicting a high risk of all-cause mortality. AUC: area under the receiver operating characteristic curve; Cys-C: cystatin C; CI: confidence interval.

The association between levels of Cys-C concentration on a continuous scale and the risk of all-cause mortality was slow rising shaped (Figure 6). According to receiver-operating characteristic curve, baseline serum Cys-C concentration was moderately capable at identifying patients with a long-term risk of all-cause mortality (AUC = 0.700; 95% CI: 0.617–0.784, *p* = 0.000; Figure 7).

## Discussion

In this study of 1107 individuals from a large-scale multicenter prospective cohort, we found that OSA severity was positively correlated with serum Cys-C concentration. This study also showed a slow rising curved association between levels of serum Cys-C and the risk of MACE and all-cause mortality, with high levels associated with a significantly increased risk. Baseline serum Cys-C levels were moderately capable of identifying patients with a long-term risk of MACE and all-cause mortality analyzed by the receiver-operating characteristic curve.

Cys-C belongs to the housekeeper gene (Zou et al., 2017), which can be transcribed and expressed at a constant rate in almost all nucleated cells. The serum Cys-C concentration is less affected by factors such as sex, age, inflammation, muscle mass, and drugs (Deveci et al., 2013). Recently, Cys-C has been considered as a sensitive indicator of early renal function impairment, which can be freely filtered by glomeruli and reabsorbed by near-curved tubules and rapidly catabolized (Cai et al., 2012; Oz-Sig et al., 2020). Studies showed that patients with sleep disorders such as OSA had significantly higher serum Cys-C levels. Besides, Cys-C levels could be reduced by continuous positive airway pressure (CPAP) treatment (Zhang et al., 2014). The mechanism of elevated Cys-C induced by OSA may include (Yang et al., 2015; Kimura et al., 2019; Yamagata, 2020): 1) intermittent hypoxia causes kidney damage by affecting sympathetic excitability, renal blood flow, glomerular filtration rate, etc. 2) hypoxemia causes oxidative stress and inflammatory response, which further result in abnormal cytokine secretion; and 3) hypoxemia and sleep disorder lead to damage of neuronal cells and increase the secretion of Cys-C. Thus, serum Cys-C concentration is expected to become a novel marker for evaluating the severity and therapeutic effect of patients with OSA.

To our knowledge, this is the first study to explore the relationship between OSA severity and baseline levels of serum Cys-C in a large-scale older population. In a previous study, a positive correlation was found between Cys-C levels and respiratory disturbance index (RDI) and T90, and a negative correlation was found between Cyst C levels and MSpO_2_ in middle-aged patients with OSA without comorbidities (Archontogeorgis et al., 2016). Another study showed that serum Cys-C levels were associated with T90 and LSpO_2_ during sleep in otherwise healthy patients with OSA (Su et al., 2021b). The present study found that AHI and ODI had a dose–response positive relationship with serum Cys-C concentration, while LSpO_2_ was negatively correlated in older patients with OSA without renal dysfunction. PSG is currently the gold standard for the diagnosis of OSA in which AHI is the most commonly used quantitative indicator of OSA severity (Berry et al., 2012). These results indicate that serum Cys-C concentration could be used as a surrogate marker to evaluate OSA severity in older patients without renal dysfunction.

Our previous study showed higher levels of baseline serum cystatin C associated with an increased risk of long-term stroke in elderly patients with OSA. However, The detailed associations between each levels of serum Cys-C and clinical outcome were not evaluated on a continuous scale (Mitaki et al., 2011). As an important finding, the present study further showed a slow rising curved association between levels of serum Cys-C and the risk of long-term cardiovascular outcomes (a composite of cardiovascular death, MI, and hospitalization for unstable angina or heart failure) and all-cause mortality during follow-up. Previous studies have shown that elevated serum Cys-C concentrations are associated with cardiovascular disease risk factors such as obesity and smoking (Angelidis et al., 2013). Cys-C plays an important role in vascular wall matrix remodeling through the balance of proteolytic and antiproteolytic activities in the arterial wall (Ruiz-Salas et al., 2014). The disturbance of vascular wall remodeling results in the pathogenesis of atherosclerotic plaque rupture, and aneurysm formation. A clinical study revealed that Cys-C values were independently associated with coronary artery calcium and coronary disease (Kiyosue et al., 2010). Similar findings were reported by Kiyosue et al. (Salgado et al., 2013) who found that the serum concentration of Cys-C correlates with the severity of coronary artery disease in patients without CKD. However, negative results were also found in other studies, which may be due to the dynamic changes of Cys-C in different pathophysiological processes of diseases (Ryan et al., 2015). In the present study, we found a slow rising curved association between levels of serum Cys-C and the risk of MACE and all-cause mortality. In this large-scale cohort, patients with the highest levels of Cys-C significantly increased their risks of long-term cardiovascular outcomes and all-cause mortality after adjustment for potential confounders. Baseline serum Cys-C concentration showed that it is moderately capable of identifying patients with a long-term risk of MACE and all-cause mortality. Thus, the present study highlighted the importance of high serum Cys-C as an important predictor of long-term clinical adverse outcomes in older patients with OSA. The degree of dyspnea in clinical manifestations and symptoms of OSA, such as daytime sleepiness and obesity, is not prominent in older people ^42^. It is more important to explore the sensitive indicators of poor prognosis in older patients with OSA. Besides, Cys-C is relatively stable in peripheral blood. According to our data, Cys-C may be a novel sensitive marker to determine the risk of long-term cardiovascular outcomes and mortality in older patients with OSA.

### Strengths and limitations

This study has two main highlights. Our study confirms the correlation between OSA severity and serum Cys-C concentration in older Asian patients with OSA. Another important finding is that serum Cys-C may be an important predictor of long-term cardiovascular outcomes and mortality in older patients with OSA. This research, however, is subject to several limitations. First, although this was a multicenter, prospective study, the study population was primarily recruited from Asian patients, which may result in unclear generalizability of the findings to non-Asian patients. Second, patients’ sleep data were collected at baseline. This is true for dynamic assessment of sleep parameters because the severity of OSA may change during the follow-up period. Third, according to our research purpose, stroke was not in the definition of MACE, given the known relationship between the Cys-C and stroke. In the future, more large-scale studies should be conducted to verify these issues. However, these limitations do not negate the value of our study.

## Conclusion

OSA severity was positively correlated with serum Cys-C concentration. Also, high levels of Cys-C were associated with increased risks of MACE and all-cause mortality in older OSA individuals. Baseline serum Cys-C levels exhibited moderately good capability at identifying patients with a long-term risk of MACE and all-cause mortality, independent of the potential risk factors. This finding, if confirmed in more studies, reveals that high baseline serum Cys-C levels should be considered as a therapeutic target in older patients with OSA and that monitoring serum Cys-C may be beneficial to the favorable prognosis of these patients.

## Data Availability

The raw data supporting the conclusions of this article will be made available by the authors, without undue reservation.
