# Supplementary material for: Association between Serum Cystatin C levels and long‐term cardiovascular outcomes and all-cause mortality in older patients with obstructive sleep apnea
Source: Front Physiol. 2022 Aug 31;13:934413. doi: 10.3389/fphys.2022.934413 (PMC9471320; doi:10.3389/fphys.2022.934413)
Supplement: Supplementary file 1 [file Image1.pdf]

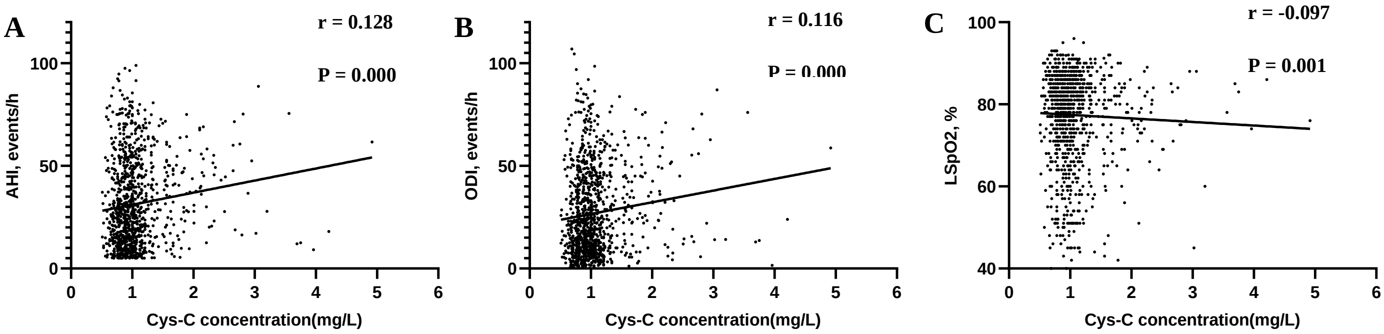

Figure S-1: Correlation between (A) AHI, (B) ODI, and (C) $LSpO_2$and serum Cys-C. AHI: the apnea-hypopnea index; ODI: the oxygen desaturation index; MSpO2: the mean pulse oxygen saturation; LSpO2: the lowest pulse oxygen saturation.
